# Chemoprevention of 4-NQO-Induced Oral Cancer by the Combination of Resveratrol and EGCG: In Vivo, In Silico and In Vitro Studies

**DOI:** 10.3390/cancers18071098

**Published:** 2026-03-28

**Authors:** Adeoluwa Adeluola, Lukmon M. Raji, Saroj Sigdel, Abu Syed Md Anisuzzaman, Md. Shamim Hossain, A. R. M. Ruhul Amin

**Affiliations:** 1 Department of Pharmaceutical Sciences, Marshall University, Huntington, WV 25755, USA; adeluola.1@osu.edu (A.A.); raji1@marshall.edu (L.M.R.); 2 Division of Pharmaceutics and Pharmacology, College of Pharmacy, The Ohio State University, Columbus, OH 43210, USA; 3 Department of Medicine, University of Tennessee Health Science Center, Memphis, TN 38163, USA; 4 Department of Pathology, School of Medicine, Marshall University, Huntington, WV 25755, USA; sigdel@marshall.edu; 5 Winship Cancer Institute of Emory University, Atlanta, GA 30322, USA; abu.anisuzzaman@flcancer.com; 6 Institute of Rheological Functions of Food, Hisayama-machi, Kasuya-gun, Fukuoka 811-2501, Japan; shamim@rheology.po-jp.com

**Keywords:** chemoprevention, oral cancer, 4-NQO, green tea, resveratrol, RNASeq, bioinformatics

## Abstract

**Background:** Squamous cell carcinoma of head and neck (SCCHN) is a devastating disease with high morbidity and mortality and the 6th most common cancer worldwide. The 5-year relative survival for advanced-stage disease is below 50%, stressing the need for chemoprevention. In the current study, we investigated the chemopreventive efficacy of the combination of resveratrol and epigallocatechin gallate (EGCG). **Methods:** We used the 4-Nitroquinoline 1-oxide (4-NQO)-induced oral carcinogenesis model. C57BL/6 mice were exposed to drinking water containing 4-NQO for 10 weeks. From week 11, mice were treated with vehicle, resveratrol, EGCG and their combination until week 22. RNASeq, qPCR and in silico analysis were performed identifying differentially expressed genes and enriched pathways. **Results:** Resveratrol alone and in combination with EGCG significantly inhibited the number of visible lesions, whereas the number of microscopic lesions and lesion areas were significantly inhibited only by the combination. The expression of Ki-67 was also significantly inhibited in resveratrol and combination groups. Growth differentiation factor 15 (*GDF15*), Activation transcription factor 3 (*ATF3*) and several other genes associated with xenobiotic metabolism as significantly upregulated genes, with *GDF15* being the most upregulated one. Furthermore, hallmarks of xenobiotic metabolism and several other anticancer pathways were enriched after treatment with resveratrol and the combination. **Conclusions:** Our data strongly demonstrate the chemopreventive potential of the combination of resveratrol and EGCG and pave the way for further clinical developments.

## Simple Summary

Chemoprevention is an approach to reduce the risk (prevent, reverse or slowdown) of invasive cancer development and progression by using chemicals or drugs. Natural dietary compounds present in fruits, vegetables and spices have been extensively studied for this purpose. In the current study, we have investigated the chemopreventive potential of the combination of natural compounds, EGCG (present in green tea) and resveratrol (present in grapes and red wine) using an oral cancer model in mice. Our results demonstrate that the combination of these two compounds significantly prevents or slows down oral cancer development in mice tongues. Cell culture studies reveal that these compounds activate genes which are essential for mitigating oxidative stress and/or inactivating xenobiotics.

## 1. Introduction

Squamous cell carcinoma of head and neck (SCCHN), tobacco-, alcohol- and human papillomavirus (HPV)-associated cancer of the oral cavity, pharynx, and larynx, is the 6th most common cancer, with about 72,680 projected new cases and about 16,680 deaths in 2025 in the United States of America [1]. SCCHNs are devastating cancers with high rates of morbidity and mortality; the 5-year relative survival for all stages is 68.5% and below 50% for advanced-stage disease [2]. While the US has seen a consistent decline in smoking rates, the use of smokeless tobacco is increasing, which also causes SCCHN [3,4]. SCCHN progresses through various premalignant stages such as hyperplasia, mild, moderate and severe dysplasia and carcinoma in situ before being transformed into malignant carcinoma, thus providing enormous opportunities for intervention at the premalignant stage, a strategy known as chemoprevention [5]. Implementation of a successful chemopreventive agent can save thousands of lives from SCCHN. However, no such approved agent/regimen is currently available for SCCHN.

The purpose of chemoprevention is to reduce the risk of cancers in normal individuals who are at high risk of developing invasive cancers. For cancer patients, certain levels of toxicity are acceptable because the potential benefits outweigh the risks of toxicity of treatment. However, toxicity is a major ethical concern for individuals who are at higher risks (smokers, HPV or hepatitis B virus infections) or with premalignant lesions/precancers, which in many cases are reversible without treatment. For this reason, natural dietary compounds present in vegetables, fruits and spices have drawn a great deal of attention for cancer chemoprevention, including the chemoprevention of SCCHN [6,7,8,9,10]. Resveratrol and epigallocatechin gallate (EGCG) are two natural compounds extensively studied for chemoprevention. EGCG is the major polyphenol present in green tea while resveratrol is a phytoalexin present in grapes, raspberries, blueberries, mulberries and peanuts. Both EGCG and resveratrol exhibit promising chemopreventive efficacy in vitro and in animal models against various cancers, including SCCHN. Resveratrol prevented DMBA-induced oral carcinogenesis in a hamster cheek pouch model and 4-NQO-induced oral tumorigenesis in mice, respectively [11,12]. EGCG restricts (slows down) the development of N-nitrosodiethylamine-induced tongue and liver carcinogenesis in mice [13]. Based on epidemiology, in vitro cell culture and in vivo animal studies, both EGCG and resveratrol were advanced to be tested in clinical trials. The safety of these agents has been well-established through these clinical trials. In a phase I trial, dose levels of 0.5 to 5.05 g/m^2^ qd and 1.0 to 2.2 g/m^2^ tid of green tea extract (GTE) were tested in 49 patients. Oral GTE at these doses can be taken safely for at least 6 months [14]. The side effects are mostly caffeine-related. In another phase II trial, GTE at doses of 500 mg/m^2^, 750 mg/m^2^, or 1000 mg/m^2^ or placebo TID were administered to patients with high-risk oral premalignant lesions for 12 weeks and were found safe [15]. Green tea preparations were also found safe in other clinical trials [16,17]. Although impressive, the results demonstrate that EGCG alone may not be sufficiently active for effective chemoprevention of SCCHN, and the combination of EGCG with other natural or synthetic agents has been suggested [14,15,18]. In fact, green tea polyphenon E in combination with erlotinib showed a high rate of pathologic response and excellent cancer-free survival in patients with advanced premalignant lesions of the oral cavity and larynx [19].

The anticancer efficacy of resveratrol against SCCHN and other cancers has been demonstrated by multiple in vitro and in vivo studies using cell lines and animal models [20,21,22,23,24]. Similarly, the safety of resveratrol has been supported by human clinical trials, which showed that oral doses of up to 5 g/day are well-tolerated [25,26]. In contrast to safety and efficacy of in vitro and animal models, resveratrol is a Multidrug Resistance 1 (MDR1) efflux pump substrate and undergoes extensive first-pass metabolism in the intestine and liver, limiting its oral bioavailability to <1% [27]. Cancer patients rarely receive a single chemo drug in their treatment regimen but a combination of multiple drugs targeting different modes of action. However, the combinatorial approach has not been widely explored in chemoprevention. Since chemoprevention targets normal individuals without invasive cancer, this combinatorial intervention should preferably be non-toxic. Both EGCG and resveratrol synergize with other natural compounds [28]. For example, the combination of EGCG and luteolin exhibited enhanced anticancer activities against SCCHN and lung cancer models [29]. EGCG also demonstrated synergistic/enhanced activity with other compounds such as nonsteroidal anti-inflammatory drugs [30] and curcumin [31]. Resveratrol also increased the efficacy of other compounds such as Actinomycin D [32], EGCG [33], curcumin [34] and quercetin [35]. However, the combination of EGCG and resveratrol has never been explored in a 4-NQO-induced oral cancer model. The central hypothesis is that the combination of EGCG and resveratrol more effectively prevents oral carcinogenesis than any single agent. We tested our hypothesis using a 4-NQO-induced oral carcinogenesis model, a model that has been widely used for oral cancer chemoprevention. In addition, we conducted RNASeq and subsequent gene enrichment analysis to identify major pathways affected by these compounds.

## 2. Materials and Methods

### 2.1. Chemoprevention Study

The animal study (pilot) was performed following animal protocol #751 approved by the Institutional Animal Care and Use Committee at Marshall University and reported in accordance with ARRIVE guidelines. Fifty (50) 6-week aged C57BL/6 female mice were purchased from the Hilltop Lab Animal, Inc. (Scottdale, PA, USA). After one week of acclimation at the Marshall University Animal Research Facility, mice were divided into two groups: non-carcinogen (*n* = 10) and carcinogen (*n* = 40). Mice in the carcinogen group received 4-NQO-containing water (50 µg/mL) for 10 weeks. The 4-NQO-containing water was changed weekly. One mouse was lost during carcinogen treatment due to an unknown reason. At week 11, mice in the carcinogen group were randomized into four groups: vehicle (*n* = 9), resveratrol (*n* = 10), EGCG (*n* = 10), combination (*n* = 10, resveratrol + EGCG). Each mouse was orally administered with vehicle, EGCG (30 mg/kg), resveratrol (30 mg/kg) or a combination of EGCG (30 mg/kg) and resveratrol (30 mg/kg), 5 days a week until 22 weeks. The doses of EGCG and resveratrol were selected based on previous studies [33,36]. 50% sweetened condensed milk was used as vehicle for drug delivery, which enabled micropipette-guided drug administration, thus significantly reducing gavage-related stress [37,38,39]. Mice in the non-carcinogen group received normal drinking water and no drug treatment throughout the entire experiment. One mouse in the vehicle group died at week 20 due to weight loss during the weekend and we were unable to collect any data. At the end of the study, mice were euthanized by CO_2_ (30–70% chamber volume/minute), the number of visible lesions was counted, tongue tissues were harvested and immediately fixed in 10% neutral buffered formalin (Z-Fix, Anatech Ltd., Battle Creek, MI, USA). Randomization and letter coded grouping were done by Dr. Amin. He also made the drug dispersions with letter coding. The students who administered the drugs were kept blind about the coding. Then, 3 µm sections (slides) were made from paraffin-embedded tissue blocks after horizontally cutting tongue tissues into three equal parts. The slides were stained with H&E and scanned using Nano Zoomer 2.0-HT (Hamamatsu, Japan). Aperio ImageScope 12.4.6 (Leica Biosystems, Buffalo Grove, IL, USA) was used for image analysis. Microscopic lesion numbers were counted using a microscope. Openlab RRID:SCR_012158 ((PerkinElmer, Waltham, MA, USA), a modular imaging software package was used to determine tumor area as previously described [40]. One mouse each from the EGCG and resveratrol group was omitted from digital analysis due to excessive folding during processing. A board-certified pathologist examined the H&E slides to confirm the grades of major lesions. The experimental design is illustrated in Supplementary Figure S1.

### 2.2. Cell Lines

As previously described, a well-characterized cell line panel comprising one malignant (MDA686TU/686Tu), one premalignant (MSK-LEUK1/MSK) and one normal human oral keratinocytes (HOK) was collected from reliable sources and used for the study [29,41]. These cell lines were grown in DMEM/F12 (Gibco, Grand Island, NY, USA) supplemented with 10% FBS (Gibco, Grand Island, NY, USA), keratinocyte basal medium (Lonza, Portsmouth, NH, USA) and oral keratinocyte medium (ScienCell, Carlsbad, CA, USA), respectively. MDA686TU and MSK-LEUK1 were procured from the original generator Dr. Peter G. Sacks’s laboratory (New York University College of Dentistry, New York, NY, USA). The cells were authenticated immediately after receiving through genomic short tandem repeat profiling at the Emory University Integrated Genomics Core (EIGC). HOK cells were purchased from ScienCell Research Laboratories (Cat. #2610; Carlsbad, CA, USA) and were grown and maintained according to the protocol provided. All cells were incubated at 37 °C and in a 5% CO_2_ humidified environment. Media was changed or sub-cultured based on confluency.

### 2.3. Immunohistochemistry Assay

Immunohistochemistry (IHC) was performed on 3 μm formalin-fixed, paraffin-embedded (FFPE) tongue sections following previously established protocols [29]. Briefly, slides were deparaffinized in xylene and rehydrated through a graded alcohol series. Endogenous peroxidase activity was quenched using 3% hydrogen peroxide. For antigen retrieval, sections were heated in 1X citrate buffer (pH 6.0) via microwave and cooled to room temperature. After blocking for 10 min with the blocking buffer (normal horse serum, 2.5%) provided in the kit (Vectastain Kit, PK7800, Vector Laboratories, Newark, CA, USA), slides were incubated overnight at 4 °C with anti-Ki-67 primary antibody (1:400; ab15580). Following washing for 5 min with wash buffer (TBST), slides were incubated with secondary antibody (provided with the kit) for 30 min. After washing for 5 min and 3,3-diaminobenzidine (DAB) visualization, sections were counterstained with hematoxylin. Ki-67 expression was quantified at 200× magnification by averaging the ratio of positive cells to total cells across five random fields.

### 2.4. Western Blotting

To prepare whole-cell lysates, cells were washed with ice-cold PBS and incubated for 1 h with a lysis buffer containing 1M Tris-HCl, 5M NaCl, 20% sodium azide, Na-deoxycholate, Igepal, and 20% SDS, supplemented with 10 µL/mL protease inhibitors (Thermo Scientific, Waltham, MA, USA). Protein concentrations were quantified using a commercial assay kit (Thermo Scientific, Waltham, MA, USA). Equal protein loads (20 µg) were resolved via 12% SDS-PAGE and transferred onto PVDF membranes (BIO-RAD, Hercules, CA, USA). Following a 1 h block in 5% non-fat skimmed milk at room temperature, membranes were incubated overnight at 4 °C with specific primary antibodies. Protein bands were visualized using the ECL Advance™ Western Blotting Detection Kit (GE Healthcare, Chicago, IL, USA) after incubation with secondary antibody for 60 min at room temperature. Mouse anti-β-actin (Sigma Life Sciences, St. Louis, MO, USA) served as the loading control.

### 2.5. RNA Extraction, RNASeq and qPCR

Total RNA was isolated from cells using the RNeasy Mini Kit (Qiagen, Germantown, MD, USA) in sextuplicate per group. Following quality control, libraries were generated from 1 μg of total RNA using the TruSeq stranded mRNA kit (Illumina, San Diego, CA, USA) and validated via the Agilent Bioanalyzer 2100 (Agilent Technologies, Santa Clara, CA, USA). Sequencing was performed on an Illumina HiSeq1500 (2 × 50 bp paired-end). Raw data were processed using bcl2fastq2 (v2.17.1.14), and Trimmomatic (v0.36) was employed to remove adapters and low-quality reads (length < 25 bp). Read quality was monitored using FastQC (v0.11.5). Trimmed reads were aligned to the GRCh38 reference genome using HISAT2 (v2.0.4), with subsequent sorting and indexing via SAMtools (v1.3.1). Differentially expressed genes (DEGs) identified using DESeq2 with an FDR threshold of 0.1. DEGs with *p* < 0.05 were considered significantly modified. The R/Bioconductor package was used to calculate the power of the RNASeq experiment to detect DEG over a range of coverage depth (ranging from 5 to 25 reads per gene), coefficient of variation (“cv”; ranging from 0.1 to 0.5) and fold change (1.5 to 2.5). The use of 6 samples per experimental group is sufficient to obtain 80% power for 2.5-fold changes at any level of cv and coverage, for 2-fold changes for all cv ≤ 0.4 except for the low coverage (<10), and for 1.5-fold changes with low cv ≤ 0.2 and coverage ≥ 15. For validation, real-time qPCR was conducted using the iTaq Universal SYBR Green One-Step Kit (BIO-RAD, Hercules, CA, USA) on a QuantStudio™ 3 system (Applied Biosystems, Foster City, CA, USA). Each 100 ng RNA sample was combined with primers (Integrated DNA Technologies; Coralville, IA, USA; Table 1). Expressions were normalized to GAPDH as an endogenous control, and all reactions were performed in triplicate. Relative expression levels were calculated using the 2^−ΔΔCt^ method as described previously [42].

### 2.6. Bioinformatics Analysis

Bioinformatic analyses were conducted entirely in R (version 4.x) using Bioconductor 3.17 and CRAN packages. Differential expression results (log_2_ fold change and *p*-values) derived from a DESeq2 pipeline were used to construct a Volcano plot in ggplot2, with genes exhibiting log_2_FC  >  1.5 and raw *p*  <  0.05 highlighted as significantly up- or downregulated. For pathway enrichment, genes meeting a stringent *p* ≤ 1 × 10^−16^ threshold were annotated via the org.Hs.eg.db database and subjected to over-representation analysis against Reactome pathways using ReactomePA. The top enriched pathways were displayed as bar-plot and dot-plot. In parallel, hallmark gene-set enrichment was performed by retrieving the MSigDB hallmark collection with msigdbr and conducting over-representation analysis using clusterProfiler’s enricher function (*p* < 0.05); enriched sets were visualized as both dot and bar charts to summarize pathway perturbations.

### 2.7. Dosage Information/Dosage Regimen

In vivo study: 4-NQO: 50 µg/mL in water; vehicle: 50% sweetened condensed milk; EGCG: 30 mg/kg dispersed in 50% sweetened condensed milk; resveratrol: 30 mg/kg dispersed in 50% sweetened condensed milk; combination: EGCG (30 mg/kg) plus resveratrol (30 mg/kg) dispersed in 50% sweetened condensed milk. Each mouse was fed with 100 μL dispersion using pipette. Mice easily swallowed drug dispersion.

RNASeq: Resveratrol: 15 μM/L (20 mM/L stock in DMSO); EGCG: 40 μM/L dissolved in water; combination: 15 μM/L resveratrol plus 40 μM/L EGCG.

qPCR and Western blotting: Resveratrol: 20 μM/L (20 mM/L stock in DMSO); EGCG: 40 μM/L dissolved in water; combination: 20 μM/L resveratrol plus 40 μM/L EGCG.

### 2.8. Statistical Analysis

Data are presented as mean ± standard deviation (SD) or standard error of the mean (SEM). Statistical analyses were performed using GraphPad Prism 10. A one-way analysis of variance (ANOVA) followed by Tukey’s post hoc test for multiple comparisons was used to determine statistical significance defined as *p* < 0.05. For testing normality distribution of data, a D’Agostino & Pearson Test was conducted.

## 3. Results

### 3.1. Prevention of 4-NQO-Induced Oral Carcinogenesis by the Combination of Resveratrol and EGCG

**Figure 1:**
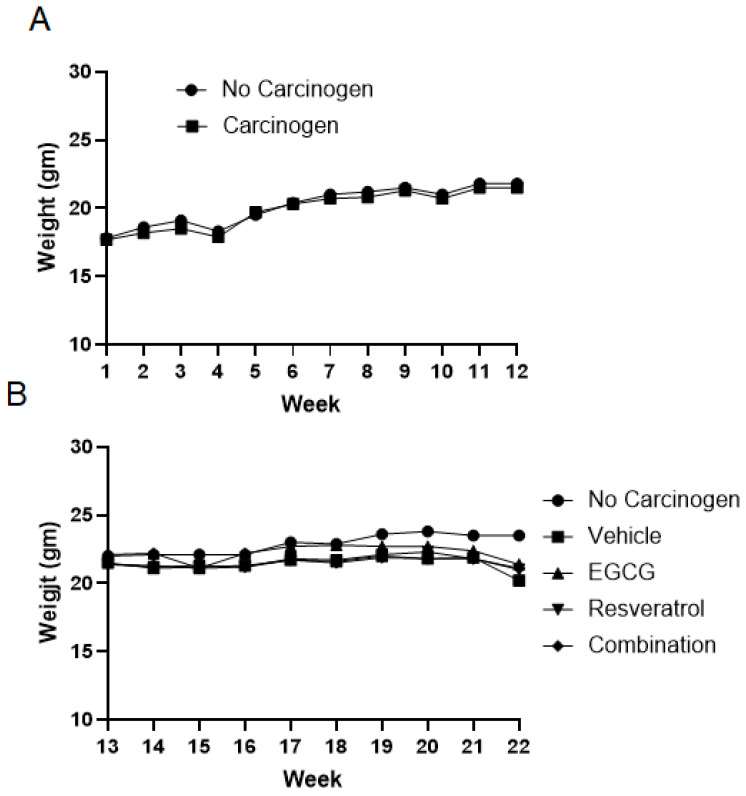
Average body weights of mice during carcinogen exposure and drug treatments: (**A**) After receiving from the vendor, the mice were divided into two groups (no carcinogen and carcinogen) and the body weight of each mouse was taken weekly. Mice were exposed to carcinogen for 10 weeks (week 2–12). (**B**) The carcinogen-exposed mice were randomized into four groups (50% sweetened condensed milk, 30 mg/kg resveratrol in 50% sweetened condensed milk, 30 mg/kg EGCG in 50% sweetened condensed milk, combination of resveratrol and EGCG) and the body weight of each mouse was taken weekly. Average body weights were plotted against time.

Although both EGCG and resveratrol have been extensively studied for the chemoprevention of various cancers as single agents, their combination has never been investigated for the chemoprevention of carcinogen-induced cancers in animal models. We have previously reported that the combination of EGCG and resveratrol is synergistic in inducing apoptosis and more strongly inhibits xenografted SCCHN tumor growths in nude mice by modulating the PI3K–AKT–mTOR pathway [33]. In the current study, we investigate the chemopreventive potential of resveratrol, EGCG and their combination against 4-NQO-induced oral carcinogenesis in mice. The experimental design is shown in Supplementary Figure S1. We monitored the body weight of mice during carcinogen exposure as well as drug treatment phases. Carcinogen exposure did not significantly affect the body weight compared to mice exposed to normal water (Figure 1A). Similarly, the body weights remained fairly stable during the drug treatment phase (Figure 1B).

**Figure 2:**
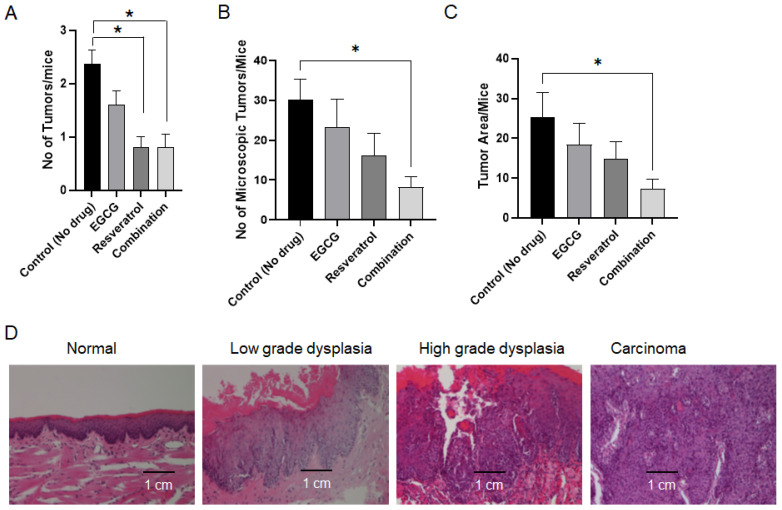
Chemoprevention of 4-NQO-induced oral carcinogenesis by resveratrol, EGCG and combination: (**A**) At the end of drug treatment, the oral cavity of each mouse was examined, and the number of visible lesions was counted. (**B**,**C**) Tumor numbers (**B**) were counted under the microscope and tumor area (**C**) was quantified using Openlab modular imaging software. All data represent mean ± SEM, * indicates statistically significant results with *p* < 0.05. (**D**) Representative images (histology) of lesions with different grades as determined by board-certified pathologist (magnification, ×200, images were reduced to 50%).

At the end of the study, the mice were euthanized and the oral cavity and tongue of each mouse were carefully examined for visible lesions. None of the mice in the non-carcinogen group developed tumors. An average of 2.3 lesions/mouse were developed in the vehicle treatment group. These numbers were 1.9/mouse, 0.8/mouse and 0.8/mouse for the EGCG, resveratrol and combination groups, respectively. Statistical analysis suggested that both resveratrol and the combination of EGCG and resveratrol significantly inhibited the number of visible lesions (Figure 2A). Moreover, the lesions in the combination group were smaller compared to the vehicle-treated group and some of the mice had no visible lesions. To digitally quantify tumor burden (number of microscopic lesions) and tumor multiplicity (microscopic lesion area in pixels) in mice, H&E-stained tongues were imaged with morphometric software to quantify the surface area composed of tumor as opposed to normal tissue of representative cross-sections of the tongue for each mouse, as previously described [40,43]. Representative images of tongues from each group are shown in Supplementary Figure S2. Results indicated that treatment of mice with the combination of EGCG and resveratrol resulted in a significant reduction in tumor burden (microscopic lesions/mouse) and multiplicity (tumor area/mouse) in mouse tongues (Figure 2B,C). A pathologist also examined the H&E-stained tongues to confirm lesions. Figure 2D shows representative normal tissues and lesions with various grades.

**Figure 3:**
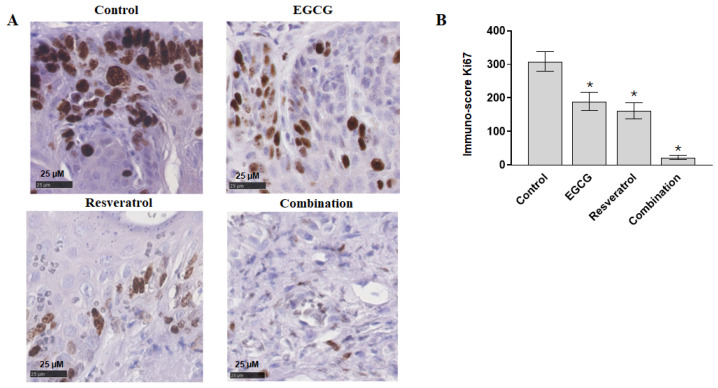
Expression of proliferation marker Ki-67 detected in lesion tissues by immunohistochemical analysis. Six randomly selected mice from each group were used for IHC staining and Ki-67 positive cells were counted from five randomly selected fields from each mouse. (**A**) Representative images are presented from each group (magnification, ×200). (**B**) Quantification of Ki-67 staining. * indicates a statistically significant difference at *p* < 0.05. *n* = six mice/group; 5 randomly selected fields.

We also measured the cellular proliferation of oral lesions by staining with Ki-67, a proliferation marker. Six randomly selected mice from each group were used for IHC staining followed by counting of Ki-67 positive cells (five randomly selected field from each slide). As shown in Figure 3, resveratrol alone and in combination with EGCG significantly inhibited the number of Ki-67 positive cells compared to untreated control. Similar to the lesion numbers, EGCG alone had no significant effect in reducing Ki-67 expression.

### 3.2. Transcriptome Analysis (RNASeq) to Identify Differentially Expressed Genes

In our previous study, we identified the AKT–mTOR pathway as a major target for the anticancer effect of the combination of resveratrol and EGCG [33]. Whole-transcriptome analysis by RNASeq is a powerful tool for identifying DEGs by drug treatments. To identify DEGs, 686Tu cells were treated with resveratrol (15 μM), EGCG (40 μM) and their combination for 24 h and total RNA was used for RNASeq. DEGs were identified as described in the methods. The number of 1.5- and 2.0-fold DEGs are listed in Table 2. The number of 2.0-fold DEGs by resveratrol was 68, of which 53 genes were upregulated, and 15 genes were downregulated. With a cut-off of 1.5-fold, this number was 445 genes, 280 upregulated, and 165 downregulated. EGCG at a dose of 40 µM had a minimal effect on gene expression, although it greatly increased the number of resveratrol-induced DEGs when combined with resveratrol, with a 1.5- and 2.0-fold cut-off; these numbers were 639 genes (358 upregulated and 281 downregulated genes) and 119 genes (83 upregulated and 36 downregulated genes). The top ten upregulated and downregulated genes in each treatment are listed in Table 3 and Table 4, respectively. *GDF15*, *AKRC1*, *AKRC2*, *AKRC3* and *LAMP3* were the most upregulated genes in the case of combination treatment. *CYP1B1*, *SPRR4* and *ALDH1A3* are among the top downregulated genes.

**Figure 4:**
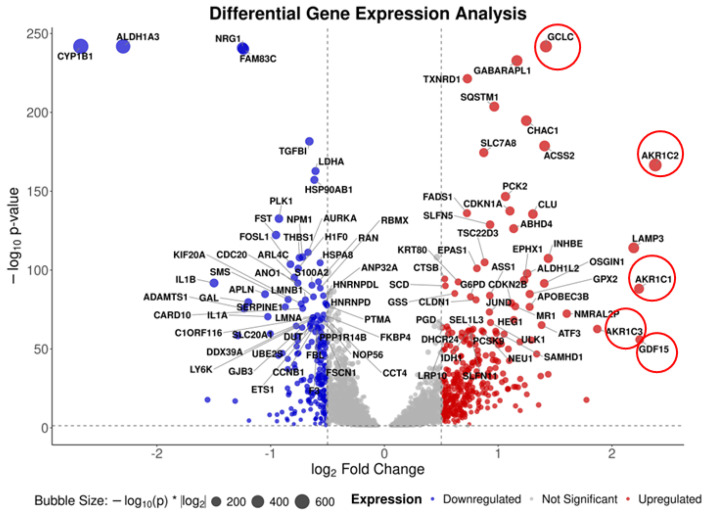
Volcano plot of differentially expressed genes from the combination group. Each point represents one of the 5187 genes surveyed in treatment versus control samples. The x-axis shows log_2_(fold change), and the y-axis shows −log_10_(*p*-value). Vertical dashed lines mark the ±0.5 log_2_FC thresholds, and the horizontal dashed line marks *p* = 0.05. Genes meeting only the *p* < 0.05 cut-off are colored gray (*n* = 4623), while those with both *p* < 0.05 and log_2_FC > 0.5 are colored red (upregulated, *n* = 360) and blue (downregulated, *n* = 204). Selected genes of interest are circled.

After cleaning the missing data within the 6020 modulated genes (untreated control versus combination), 5187 genes were used for Volcano plot analysis. The very high log_10_*p* values indicated that most of the genes were significantly changed compared with the untreated control (Figure 4).

### 3.3. Bioinformatics Analysis of Differentially Expressed Genes to Identify Affected Pathways

**Figure 5:**
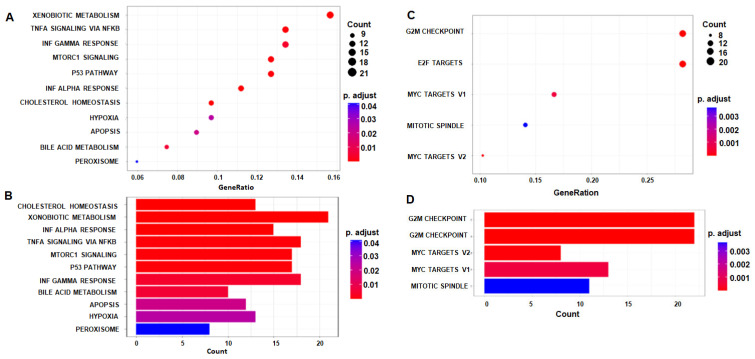
Top significantly enriched pathways after combined treatment with EGCG and resveratrol: (**A**) Dot-plot displays the top Reactome pathways enriched among genes with *p* ≤ 1 × 10^−16^, where the x-axis shows the GeneRatio (i.e., the fraction of input genes overlapping each pathway); each dot’s size corresponds to the number of overlapping genes (“Count”), and the dot color indicates enrichment significance as −log_10_(*p*-value) (darker red = more significant). (**B**) Re-plot of the same Reactome pathways as a horizontal bar chart: the x-axis represents the gene count per pathway, and bar color reflects −log_10_(*p*-value) (red → blue gradient, red = higher significance). (**C**) Dot-plot of the top hallmark gene sets (MSigDB “H”) enriched in the identical gene list using the same mapping of GeneRatio (x-axis), dot size (gene count), and dot color (−log_10_[*p*-value]) as in (**A**). (**D**) Represents those top hallmark sets as a horizontal bar-plot, with gene count on the x-axis and enrichment significance (−log_10_[*p*-value]) encoded by bar color.

To identify the most affected pathways, we conducted bioinformatics analysis/pathway enrichment using DEGs of combined treatment. Figure 5A,B shows the top upregulated pathways when plotted against gene ratio and gene count, respectively. Hallmarks of xenobiotic metabolism, cholesterol homeostasis, TNFα signaling via NFκB, mTORC1 signaling, apoptosis and p53 pathway are among the top upregulated pathways. The most downregulated pathways are hallmarks of G2M checkpoint, E2F targets, MYC targets and mitotic spindle (Figure 5C,D). Supplementary Files S1 and S2 show the upregulated and downregulated pathways and the associated genes, respectively.

### 3.4. Confirming the Expression of Selected Genes and Proteins by Real-Time qPCR and Western Blotting

**Figure 6:**
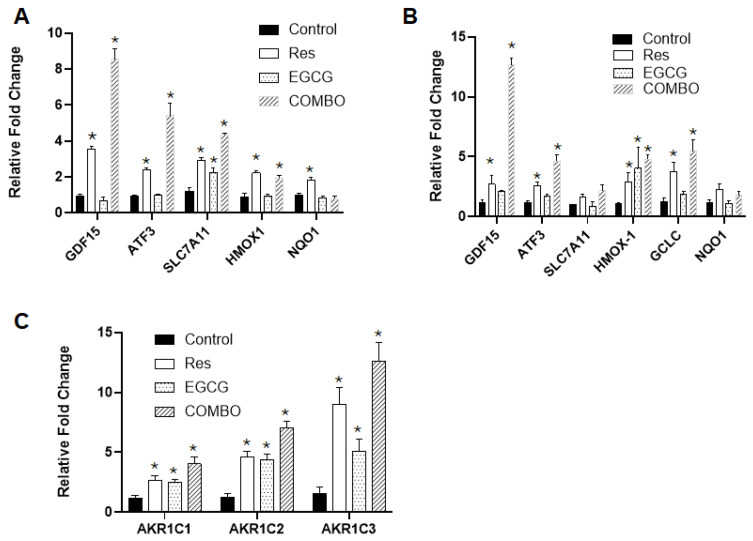
Expression of genes associated with xenobiotic metabolism and antioxidant defense: (**A**) 686Tu and (**B**) MSK cells were treated with 20 µM resveratrol, 40 µM EGCG and a combination of 20 µM resveratrol and 40 µM EGCG for 24 h. Total RNA was used for the expression of *GDF15*, *ATF3*, *SLC7A11*, *HMOX1*, *NQO1* and *GCLC* by qPCR. Error bars represent SD from triplicate treatments. (**C**) 686Tu cells were treated as above. Total RNA was used for the expression of *AKR1C1*, *AKR1C2* and *AKR1C3*. * indicates statistically significant difference (*p* < 0.05). *n* = 3 for all genes.

*GDF15* was identified as the most upregulated gene and hallmark of the xenobiotic metabolism as the top upregulated pathway. We examined the mRNA expression of some selected genes including *GDF15* and those associated with the xenobiotic metabolism pathway and activated in response to oxidative stress. As shown in Figure 6A–C, treatment with resveratrol, EGCG or the combination of resveratrol and EGCG significantly increased the expression of *GDF15*, *ATF3*, Solute Carrier Family 7 Member 11 (*SLC7A11*), Heme Oxygenase-1 (*HMOX1*), NAD(P)H Quinone Dehydrogenase 1 (*NQO1*), Aldo-Keto Reductase Family 1 Member C (*AKR1C*)*1*, *AKR1C2* and *AKR1C3* in 686Tu (malignant) and MSK (premalignant) cells, although the effects of EGCG were not significant in some cases. Although EGCG had a minimal effect on the expression of *GDF15* and *ATF3*, the combination of resveratrol and EGCG greatly increased the expression of these genes. *GDF15* is a known transcriptional target of ATF3 [44,45].

**Figure 7:**
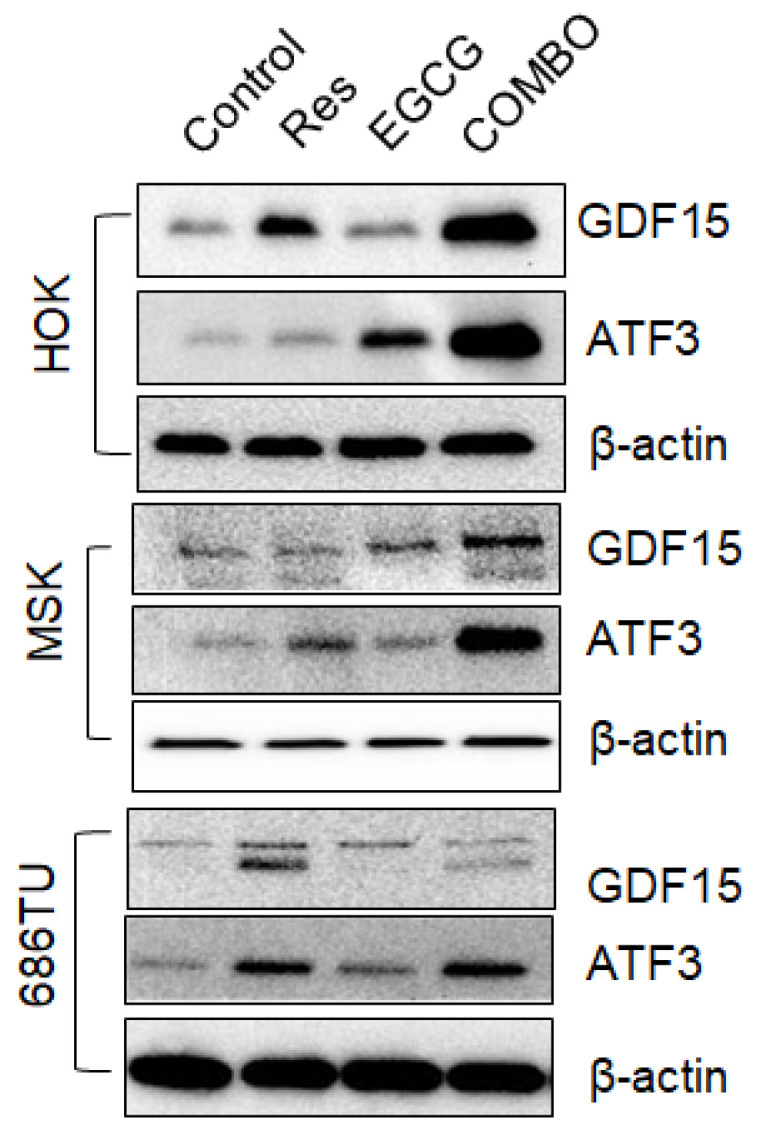
Expression of ATF3 and GDF 15 proteins: HOK, MSK and 686Tu cells were treated with 20 µM resveratrol, 40 µM EGCG and a combination of 20 µM resveratrol and 40 µM EGCG for 24 h. Whole-cell lysates were used for the expression of GDF15 and ATF3 proteins by Western blotting. The uncropped bolts are shown in Supplementary Materials.

We also examined the expression of GDF15 and ATF3 proteins in normal (HOK), premalignant (MSK) and malignant (686Tu) head and neck cell lines and found that, like mRNA expression, resveratrol, EGCG and their combination also increased the expression of GDF15 and ATF3 proteins (Figure 7).

## 4. Discussion

Although chemoprevention is a well-justified, viable option for reducing cancer burden, its success is limited, with approved chemoprevention drugs for breast cancer only. Unlike the treatment of cancer patients where the question is about life and death, toxicity is a significant concern for chemoprevention as the subjects have not yet developed invasive cancer but are at high risk. As a result, diet-derived natural agents drew much attention for chemoprevention because of their non-toxic nature and people’s acceptance. Moreover, cancer patients rarely receive single drug regimens in their treatment plan; rather, combinations of multiple modalities or agents are common. However, the combinatorial approach has not been explored well in chemoprevention. In the current study, we investigated the chemopreventive potential of the combination of two natural compounds, resveratrol and EGCG against 4-NQO-induced oral carcinogenesis in an in vivo model extensively used for testing compounds for their chemopreventive potential against oral carcinogenesis. 4-NQO is a carcinogen present in tobacco smoke and mimics the histological and biological processes of human oral carcinogenesis including the genetic and molecular changes seen in the progression from normal to mild, moderate and severe dysplasia, and from carcinoma in situ to invasive carcinoma [46,47]. Although both resveratrol and green tea preparations exhibited chemopreventive efficacy against oral carcinogenesis in animal models, GTE alone was ineffective in preventing oral cancer development in a phase II clinical trial which prompted us to explore a combinatorial approach [15]. Our findings demonstrate that, although resveratrol alone or in combination with EGCG significantly inhibited the number of visible tumors, EGCG alone had a insignificant effect, as the EGCG dose is lower than our previous in vivo doses [29,33]. However, visible lesions do not always represent the real fact, particularly, if the intervention delays carcinogenesis. In contrast, digital imaging and microscopic counting after H&E staining allows the detection of microscopic lesions. Only the combination of EGCG and resveratrol significantly inhibited the number and the area of microscopic lesions, suggesting that resveratrol alone delayed tumor development, as indicated by the increased number of microscopic lesions. The chemopreventive efficacy was also supported by Ki-67 expression. Our data are consistent with a previous study which tested the efficacy of resveratrol (AIN-76A diet supplemented with 0.25% *w*/*w* resveratrol for 8 weeks) against 4-NQO-induced oral carcinogenesis. Although the effect on tumor incidence is moderate, the multiplicity and severity of lesions were significantly inhibited [12]. An average of 4.66 and 1.33 lesions/mouse was detected in the 4-NQO and resveratrol group, respectively. In contrast, our data are inconsistent with a previous study testing the efficacy of green tea polyphenols (GTE) against 4-NQO-induced oral carcinogenesis [48]. Simultaneous treatments of rats with 4-NQO and GTE significantly reduced the number of tumors, tumor volume and SCCHN. The results were also significant when GTE treatment was given after 4-NQO exposure. The inhibition of tumor incidence is 88.75%. The inconsistency with our study is probably attributed to dosing (200 mg/kg vs. 30 mg/kg).

We also explored the cell signaling pathways modulated by these compounds and their combination through RNASeq and subsequent gene enrichment analysis to identify significant pathways and genes affected by EGCG, resveratrol and their combination. *GDF15*, a member of the TGF-β superfamily, was identified as the most upregulated gene. Accumulated evidence from several carcinogenesis models suggests that *GDF15* is expressed under oxidative stress and plays a pivotal role in the chemoprevention of intestinal cancer by sulindac [49,50], colon cancer by cox inhibitors, sulindac and celecoxib [51], and chemically induced breast cancer by the combination of celecoxib and resveratrol [52], etc. Several natural compounds induced apoptosis of head and neck cancer cell lines by upregulating the expression of GDF15 [53,54]. However, its role in oral carcinogenesis and chemoprevention is largely unknown. We confirmed the upregulation of *GDF15* mRNA and protein in normal, premalignant and malignant SCCHN by EGCG, resveratrol and their combination. Furthermore, we confirmed the upregulation of ATF3, a transcription factor that regulates *GDF15*. Further studies using *GDF15* knockout mice are required to verify the role of *GDF15* in oral carcinogenesis and chemoprevention.

The hallmark of xenobiotic metabolism was identified as one of the top significantly upregulated pathways based on gene ratio and gene count. Xenobiotic metabolism is crucial for the detoxification of foreign chemicals including carcinogens. In addition, many genes involved in xenobiotic metabolism are also involved in antioxidant defense mechanisms to protect against oxidative stress. Carcinogenesis is initiated with irreversible genetic mutations resulting from oxidative stress generated endogenously or by carcinogen exposure. 4-NQO generates oxidative stress/reactive oxygen species leading to oral and esophageal carcinogenesis [55,56,57]. Resveratrol or its combination with EGCG upregulates several antioxidant genes such as *GCLC*, *GCLM*, *NQO1*, *HMXO1*, *SLC7A11*, *CAT*, etc. Other modulated pathways include the upregulation of the p53 pathway, apoptosis pathway, interferon-α and -γ response, and the downregulation of G2/M checkpoint, E2F1 targets, MYC targets, mitotic spindle, glycolysis, mTORC1, etc. Most of these pathways are known to mediate anticancer effects or carcinogenesis and might be involved in the chemopreventive effect of these compounds.

## 5. Conclusions

Taken together, our findings demonstrate that the combination of resveratrol and EGCG prevents or delays 4-NQO-induced oral carcinogenesis, upregulates pathways which are important for xenobiotic metabolism, mitigation of oxidative stress and apoptosis and downregulates several oncogenic pathways such as mTORC1, Myc and E2F1. Although promising, the study has several limitations. Firstly, the in vivo experiment was conducted exclusively in female mice, rendering the analysis of sex-dependent effects impossible. Secondly, the RNASeq was performed using a malignant cell line. Although the expression of critical genes was further validated in normal and premalignant cells, the expression of these genes was not tested in animal tissues. Thirdly, the cell line data are simply correlative with the animal study. Finally, the effects of the carcinogen and drug treatments on major organs (toxicity) were not explored. Future studies need to focus on functional studies of the genes like *GDF15* or pathways like antioxidant and xenobiotic metabolism in the chemoprevention of oral cancer using animal models. Despite these limitations, our study opens new avenues for testing the toxicity and pharmacokinetics of these two promising natural compounds, which will ultimately pave the way for clinical trials.

## Figures and Tables

**Table 1:** Primer sequences used for RT-qPCR.

Gene	Forward	Reverse
*GAPDH*	5′-TGCACCACCAACTGCTTA-3′	5′-GGATGCAGGGATGATGTTC-3′
*GDF15*	5′-GTCCGGATACTCACGCCAG-3′	5′-TCACGTCCCACGACCTTGAC-3′
*ATF3*	5′-GTTTGAGGATTTTGCTAACCTGAC-3′	5′-AGCTGCAATCTTATTTCTTTCTCGT-3′
*SLC7A11*	5′-GGCTCCATGAACGGTGGTGTG-3′	5′-GCTGGTAGAGGAGTGTGCTTGC-3′
*HMOX1*	5′ ATTTCAGAAGGGCCAGGTGA 3′	5′ GGAAGTAGACAGGGGCGAAGA 3′
*NQO1*	5′-GTCATTCTCTGGCCAATTCAGAGT-3′	5′-TTCCAGGATTTGAATTCGGG-3′
*GCLC*	5′-GGCGATGAGGTGGAATACAT-3′	5′-GTCCTTTCCCCCTTCTCTTG-3′
*AKR1C1*	5′-GTAAAGCTTTAGAGGCCAC-3′	5′-GAGGTCAACATAATCCAATTG-3′
*AKR1C2*	5′-AACCAGGCCAGTGACAGAAA-3′	5′-AGCAGCCTGTGGTTGAAGTT-3′
*AKR1C3*	5′-CCAATGTCTCTAAAGCCAGG-3′	5′-TAGACATCAGGCAAAGCCCT-3′

**Table 2:** Numbers of significantly (*p*  <  0.05) up- and downregulated DEGs with 1.5- and 2.0-fold cut-offs.

Treatment	1.5-Fold			2-Fold		
	Total	Up	Down	Total	Up	Down
EGCG	9	3	6	3	0	3
Resveratrol	445	280	165	68	53	15
Combination	639	358	281	119	83	36

**Table 3:** Top 10 significantly (*p* < 0.05) upregulated genes based on fold change.

Rank	Resveratrol		EGCG		Combination	
	Gene	Fold	Gene	Fold	Gene	Fold
1	*AKR1C2*	5.2	*BNIP3*	1.8	*GDF15*	6.8
2	*GDF15*	4.7	*SAA1*	1.7	*LAMP3*	5.8
3	*AKR1C1*	4.7	*P4HA1*	1.3	*AKR1C2*	5.1
4	*LAMP3*	4.6	*BNIP3L*	1.3	*AKR1C1*	4.6
5	*AKR1C3*	3.7	*ARRDC4*	1.3	*CXCL8*	3.8
6	*S100P*	3.4	*RILP*	1.3	*AKR1C3*	3.7
7	*NMRAL2P*	3	*NDRG1*	1.3	*LURAP1L*	3.6
F	*INHBE*	2.7	*DCAKD*	1.3	*ATF3*	3.5
9	*TMEM125*	2.7	*MOB3A*	1.3	*SAA1*	3.3
10	*GCLV*	2.7	*SELENOO*	1.3	*ACSS2*	3.2

**Table 4:** Top 10 significantly (*p* < 0.05) downregulated genes based on fold change.

Rank	Resveratrol		EGCG		Combination	
	Gene	Fold	Gene	Fold	Gene	Fold
1	*CYP1B1*	−6.4	*SELENOW*	−3.0	*CYP1B1*	−6.9
2	*ALDH1A3*	−4.9	*CGB5*	−2.7	*SPRR4*	−5.1
3	*SPRR4*	−2.9	*CGB8*	−2.2	*ALDH1A3*	−4.9
4	*IL1β*	−2.8	*SELENOH*	−1.6	*SELENOW*	−3.2
5	*H19*	−2.5	*KRT81*	−1.6	*SPRR2G*	−3.0
6	*ALPP*	−2.4	*GPX1*	−1.5	*ALPP*	−3.0
7	*NRG1*	−2.4	*CYPA1*	−1.4	*CRCT1*	−3.0
8	*FAM83C*	−2.4	*ALDH1A3*	−1.4	*LCE3D*	−2.9
9	*ADATS1*	−2.4	*HOXC13-AS*	−1.4	*H19*	−2.8
10	*GAL*	−2.3	*ID3*	−1.4	*HIST1H4J*	−2.8

## Data Availability

The data supporting the findings of this study are available from the corresponding author upon reasonable request. Raw and processed sequencing data were deposited in the Gene Expression Omnibus (GEO) on 19 August 2025, maintained by the National Center for Biotechnology Information at the National Library of Medicine, and are available via accession number GSE305867.

## Supplementary Materials

The following supporting information can be downloaded at: https://www.mdpi.com/article/10.3390/cancers18071098/s1, Figure S1: Schema for chemoprevention study; Figure S2: Three H&E stained mice tongues from each group. File S1: Upregulated Pathways; File S2: Downregulated pathways.

## Abbreviations

The following abbreviations are used in this manuscript:

SCCHN	Squamous cell carcinoma of head and neck
GDF15	Growth differentiation factor 15
ATF3	Activation transcription factor 3
HPV	Human papillomavirus
EGCG	Epigallocatechin gallate
GTE	Green tea extract
HOK	Human oral keratinocytes
IHC	Immunohistochemistry
FFPE	Formalin-fixed, paraffin-embedded
DAB	3,3 diaminobenzidine
DEG	Differentially expressed gene
4-NQO	4-nitro quinoline oxide
SLC7A11	Solute Carrier Family 7 Member 11
HMOX1	Heme Oxygenase-1
NQO1	NAD(P)H Quinone Dehydrogenase 1
AKR1C	Aldo-Keto Reductase Family 1 Member C
