# Correction: Adeluola et al. Chemoprevention of 4-NQO-Induced Oral Cancer by the Combination of Resveratrol and EGCG: In Vivo, In Silico and In Vitro Studies. *Cancers* 2026, *18*, 1098

**DOI:** 10.3390/cancers18121971

**Published:** 2026-06-17

**Authors:** Adeoluwa Adeluola, Lukmon M. Raji, Saroj Sigdel, Abu Syed Md Anisuzzaman, Md. Shamim Hossain, A. R. M. Ruhul Amin

**Affiliations:** 1 Department of Pharmaceutical Sciences, Marshall University, Huntington, WV 25755, USA; adeluola.1@osu.edu (A.A.); raji1@marshall.edu (L.M.R.); 2 Division of Pharmaceutics and Pharmacology, College of Pharmacy, The Ohio State University, Columbus, OH 43210, USA; 3 Department of Medicine, University of Tennessee Health Science Center, Memphis, TN 38163, USA; 4 Department of Pathology, School of Medicine, Marshall University, Huntington, WV 25755, USA; sigdel@marshall.edu; 5 Winship Cancer Institute of Emory University, Atlanta, GA 30322, USA; abu.anisuzzaman@flcancer.com; 6 Institute of Rheological Functions of Food, Hisayama-machi, Kasuya-gun, Fukuoka 811-2501, Japan; shamim@rheology.po-jp.com

## Missing Citation

In the original publication [1], “Raji, L.; Afrin, R.; Amin, J.; Lamichhane, R.; Denning, K.; Amin, A. In vitro and in vivo anticancer efficacy of the combination of actinomycin D and resveratrol. *Biochem. Cell Biol.* **2026**, *104*, 1–11” was not cited. The citation has now been inserted in Introduction, paragraph 3 and should read as follows:

The anticancer efficacy of resveratrol against SCCHN and other cancers has been demonstrated by multiple in vitro and in vivo studies using cell lines and animal models [20–24]. Similarly, the safety of resveratrol has been supported by human clinical trials, which showed that oral doses of up to 5 g/day are well-tolerated [25,26]. In contrast to safety and efficacy of in vitro and animal models, resveratrol is a Multidrug Resistance 1 (MDR1) efflux pump substrate and undergoes extensive first-pass metabolism in the intestine and liver, limiting its oral bioavailability to <1% [27]. Cancer patients rarely receive a single chemo drug in their treatment regimen but a combination of multiple drugs targeting different modes of action. However, the combinatorial approach has not been widely explored in chemoprevention. Since chemoprevention targets normal individuals without invasive cancer, this combinatorial intervention should preferably be non-toxic. Both EGCG and resveratrol synergize with other natural compounds [28]. For example, the combination of EGCG and luteolin exhibited enhanced anticancer activities against SCCHN and lung cancer models [29]. EGCG also demonstrated synergistic/enhanced activity with other compounds such as nonsteroidal anti-inflammatory drugs [30] and curcumin [31]. Resveratrol also increased the efficacy of other compounds such as Actinomycin D [32], EGCG [33], curcumin [34] and quercetin [35]. However, the combination of EGCG and resveratrol has never been explored in a 4-NQO-induced oral cancer model. The central hypothesis is that the combination of EGCG and resveratrol more effectively prevents oral carcinogenesis than any single agent. We tested our hypothesis using a 4-NQO-induced oral carcinogenesis model, a model that has been widely used for oral cancer chemoprevention. In addition, we conducted RNASeq and subsequent gene enrichment analysis to identify major pathways affected by these compounds.

With this correction, the order of some references has been adjusted accordingly. The authors state that the scientific conclusions are unaffected. This correction was approved by the Academic Editor. The original publication has also been updated.
