# Supplementary material for: Chemoprevention of 4-NQO-Induced Oral Cancer by the Combination of Resveratrol and EGCG: In Vivo, In Silico and In Vitro Studies
Source: Cancers (Basel). 2026 Mar 28;18(7):1098. doi: 10.3390/cancers18071098 (PMC13072290; doi:10.3390/cancers18071098)
Supplement: Supplementary file 1 [file cancers-18-01098-s001.zip › cancers-4187024 full western blotsDensitometry_raw_EGCG_RES_data_2_23_2026.pptx]

## Slide 1
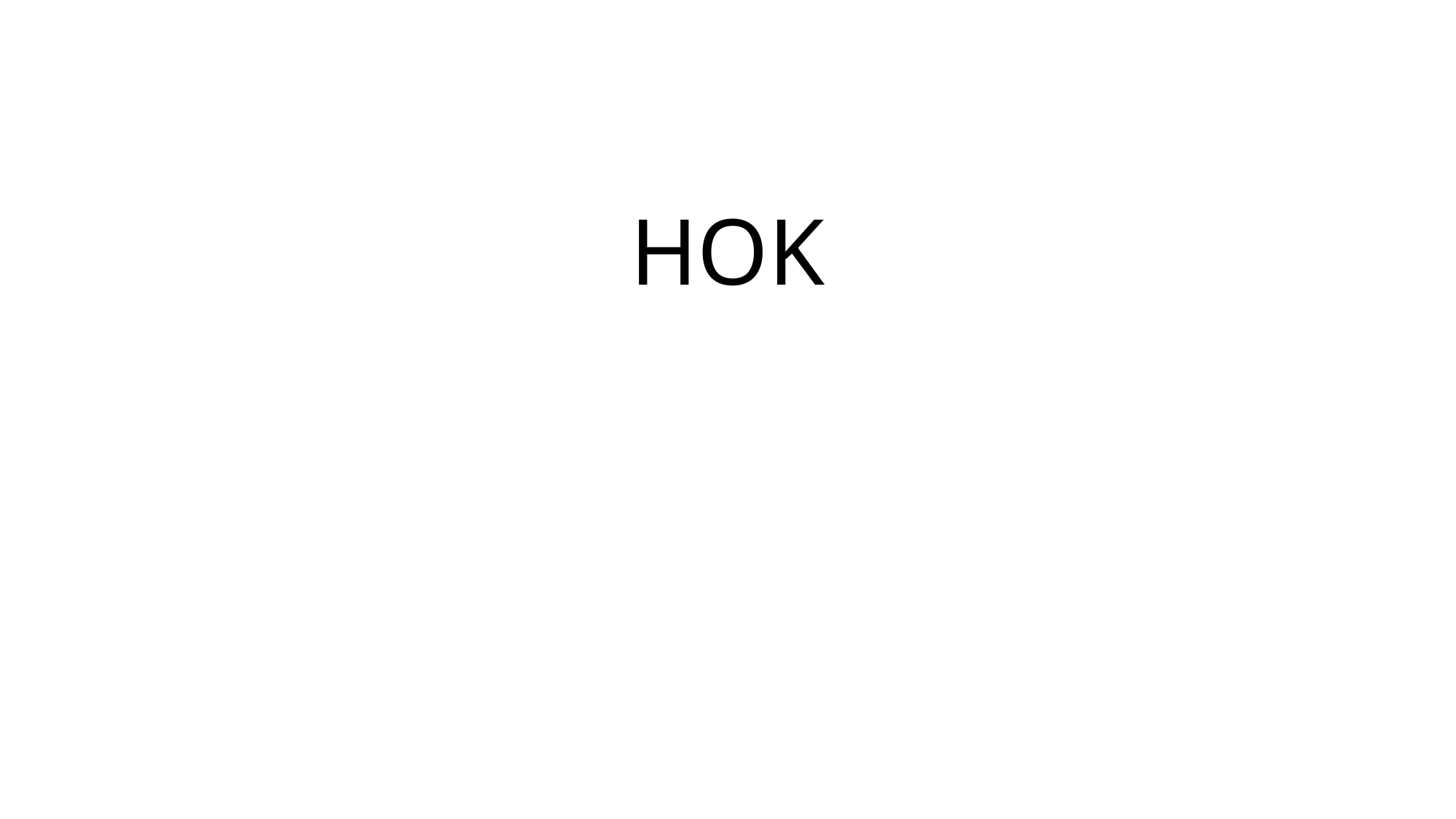

# HOK

## Slide 2
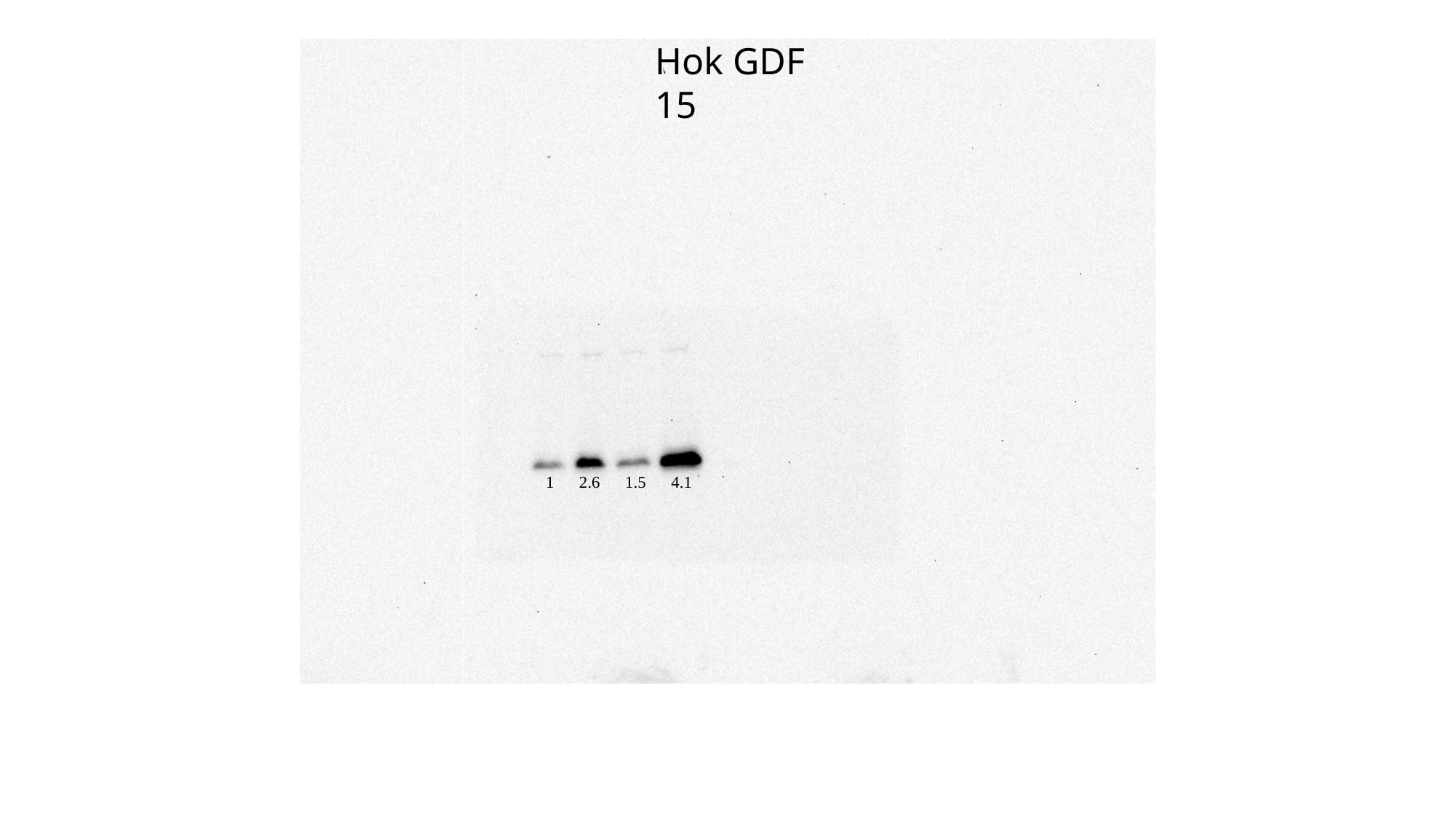

Hok GDF 15
1 2.6 1.5 4.1

## Slide 3
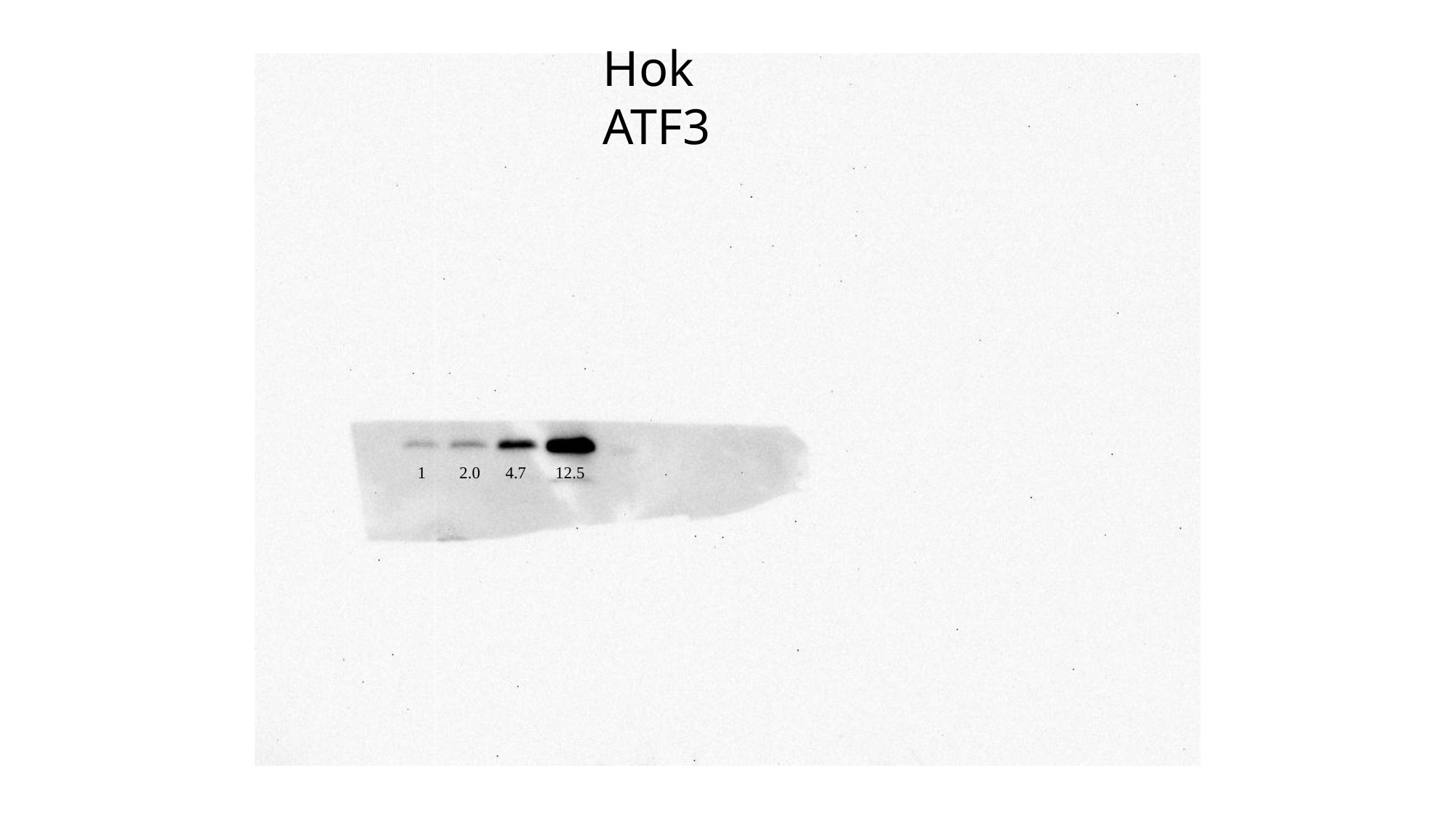

Hok ATF3
1 2.0 4.7 12.5

## Slide 4
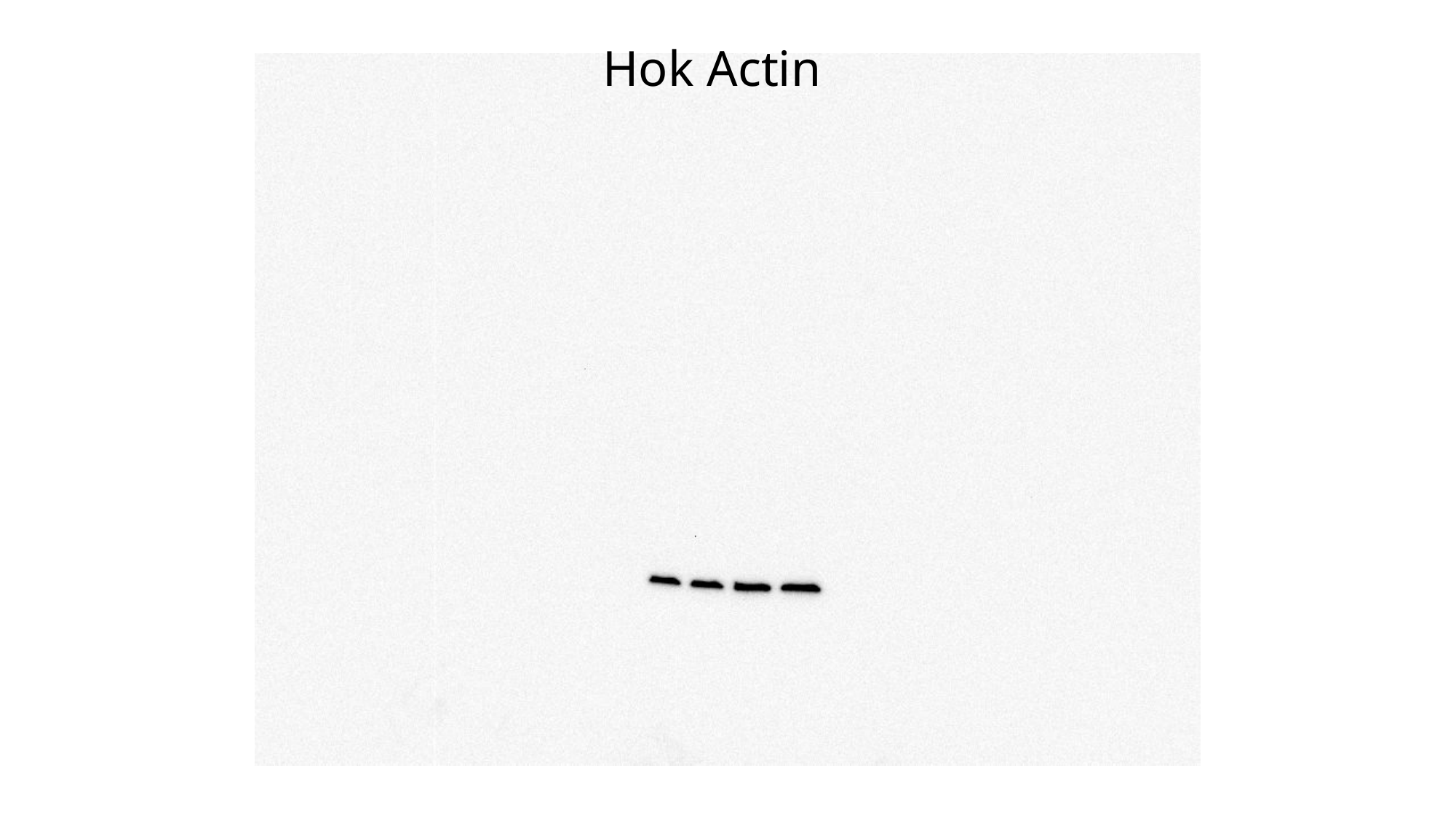

Hok Actin

## Slide 5
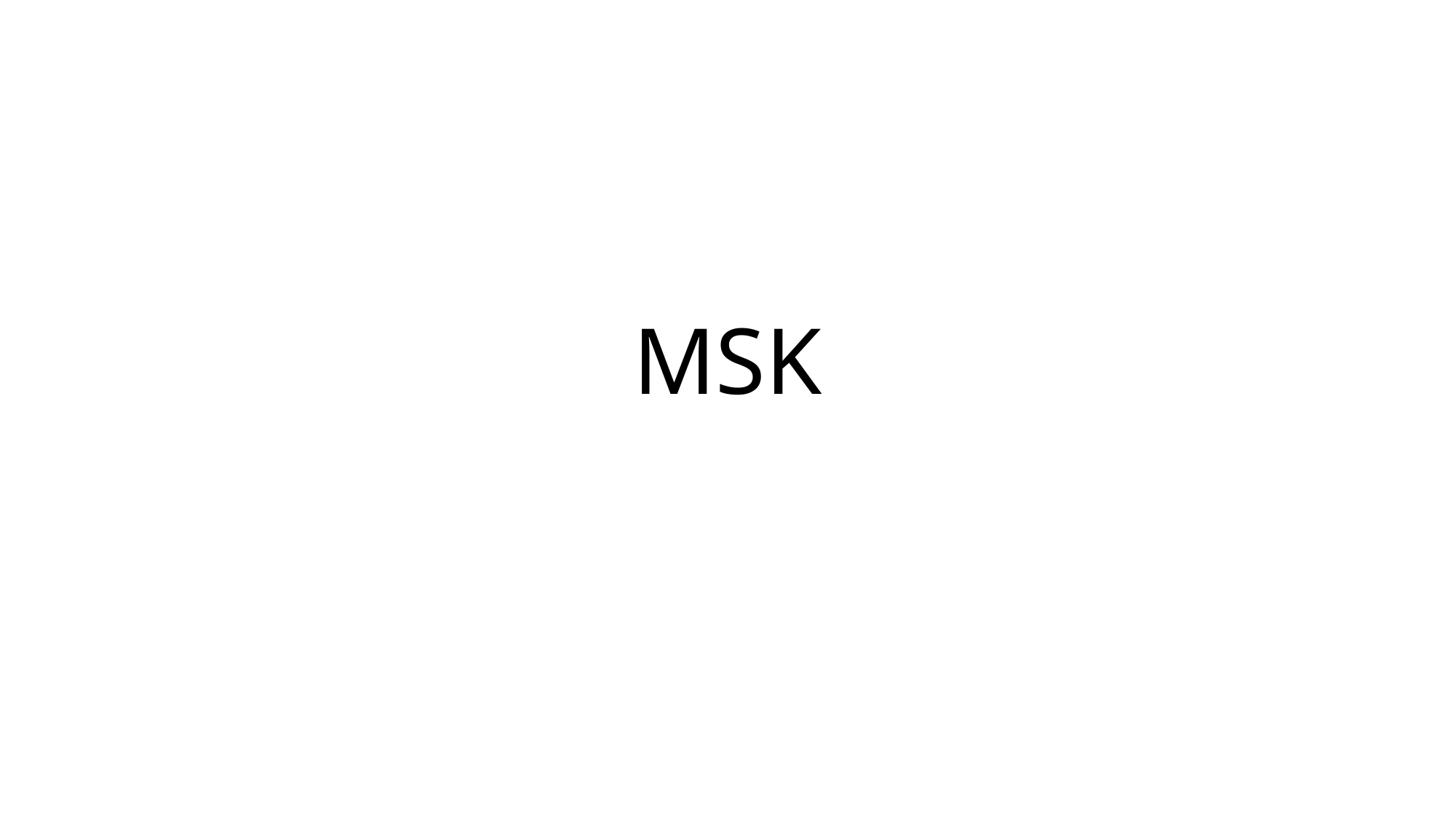

# MSK

## Slide 6
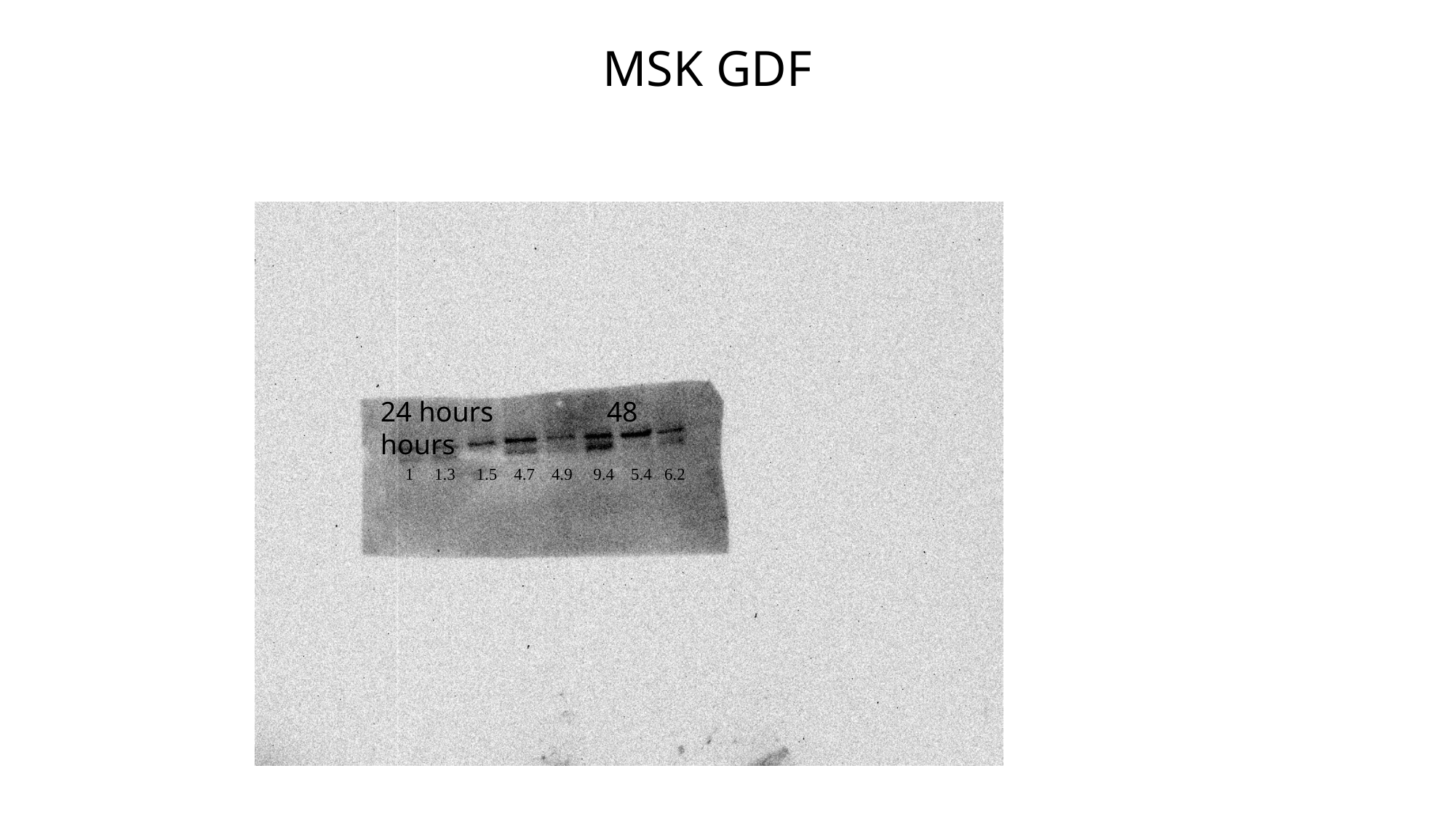

MSK GDF
24 hours 48 hours
1 1.3 1.5 4.7 4.9 9.4 5.4 6.2

## Slide 7
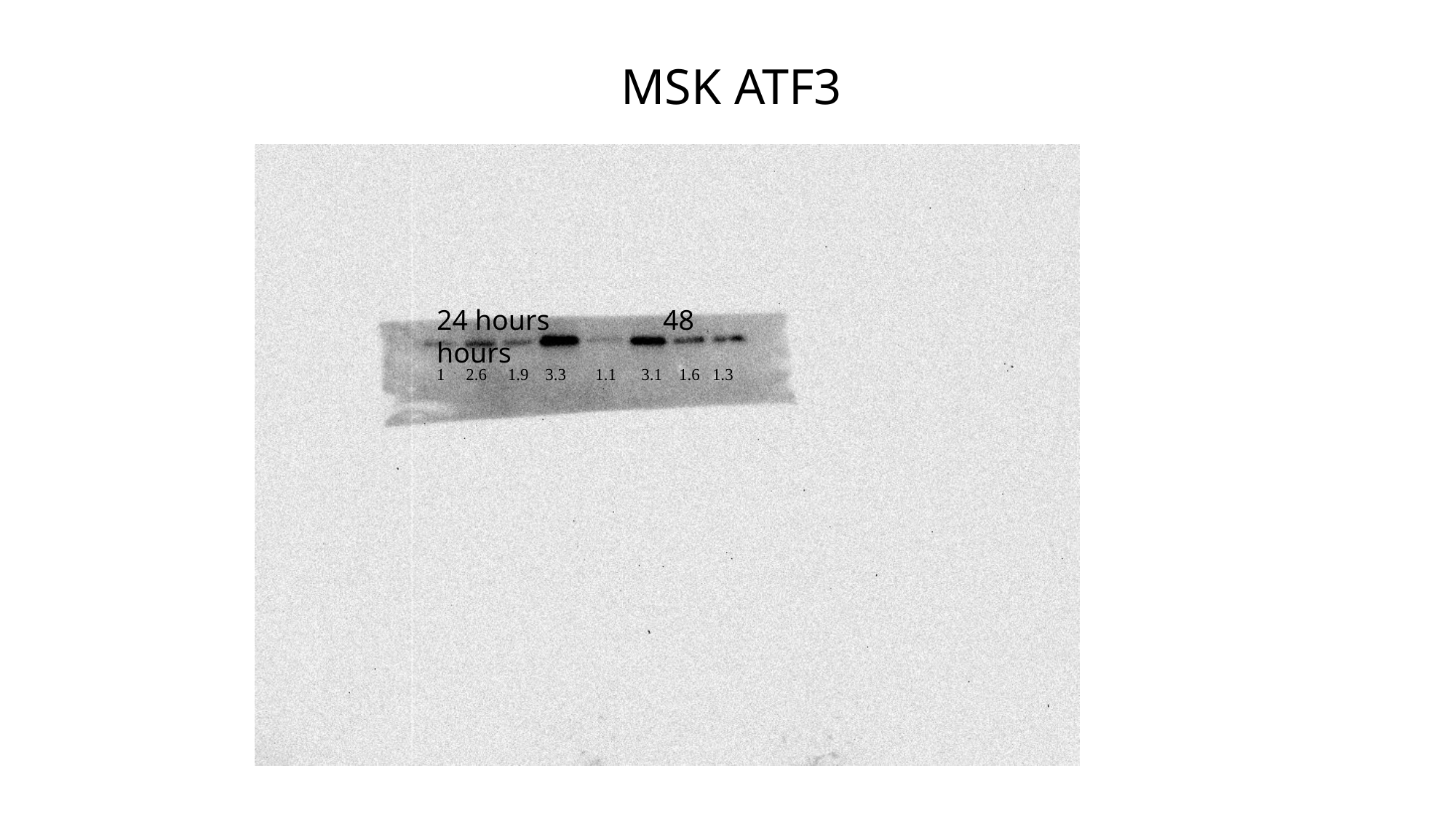

MSK ATF3
24 hours 48 hours
1 2.6 1.9 3.3 1.1 3.1 1.6 1.3

## Slide 8
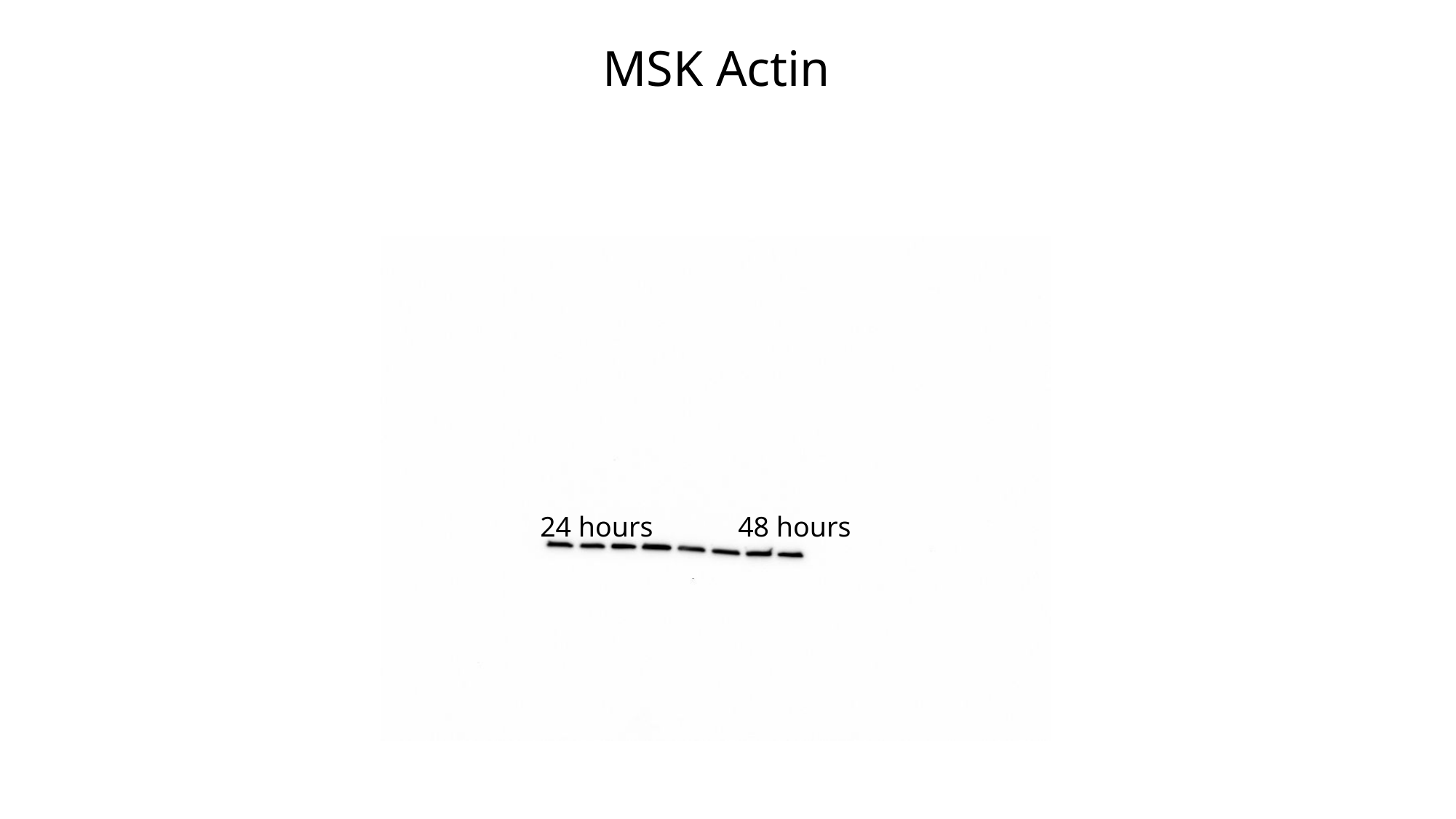

MSK Actin
24 hours 48 hours

## Slide 9
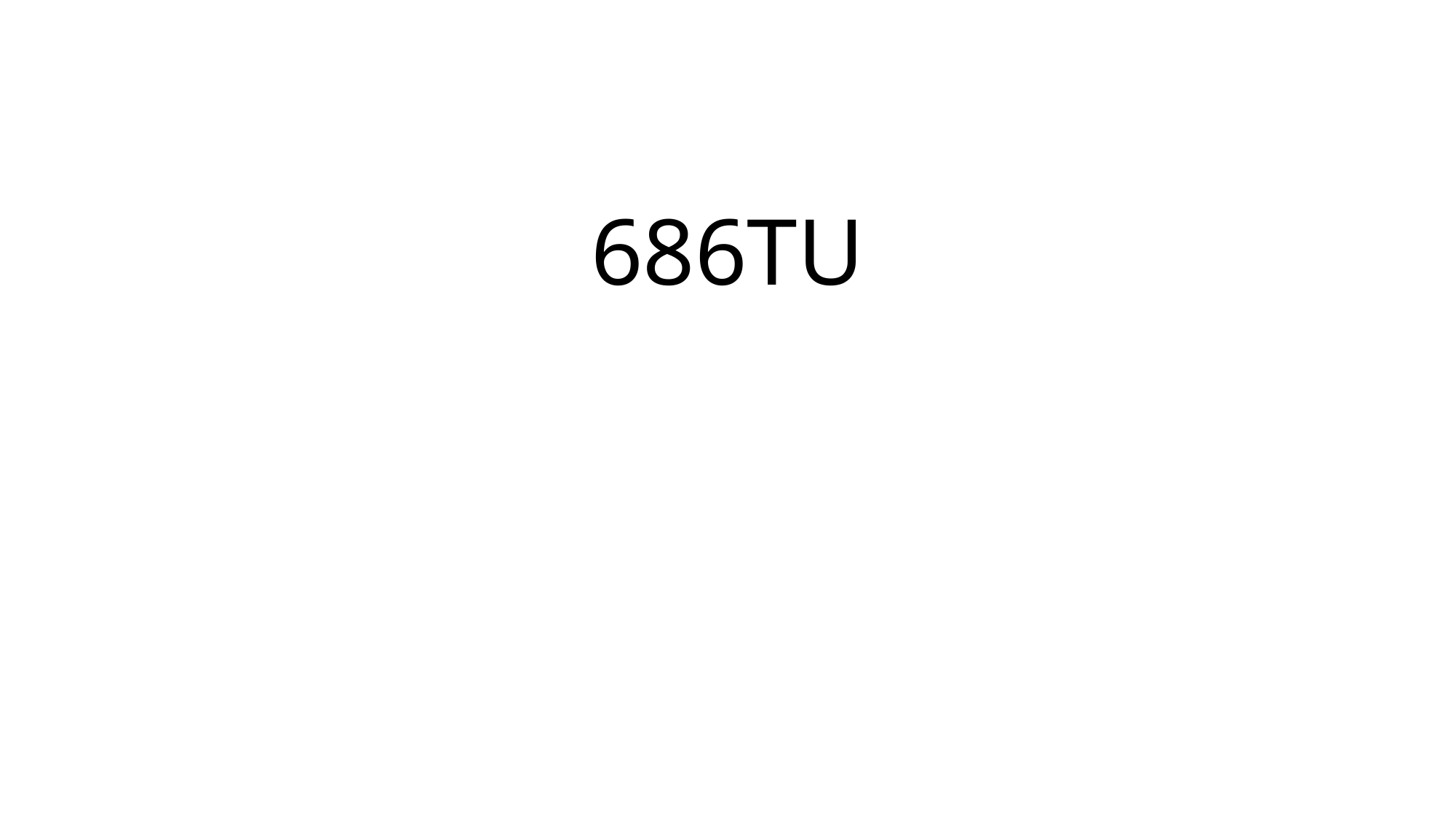

# 686TU

## Slide 10
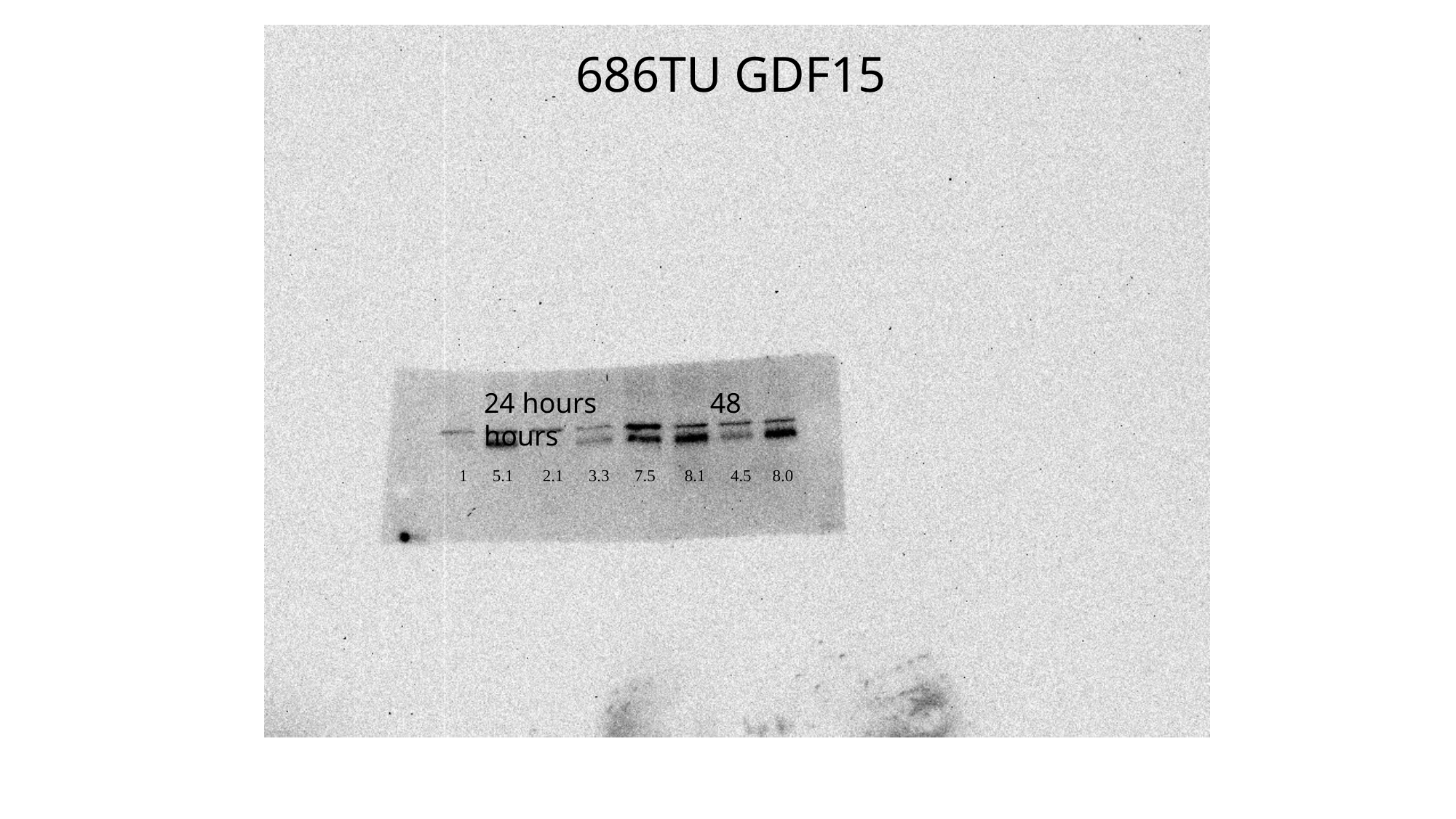

686TU GDF15
24 hours 48 hours
1 5.1 2.1 3.3 7.5 8.1 4.5 8.0

## Slide 11
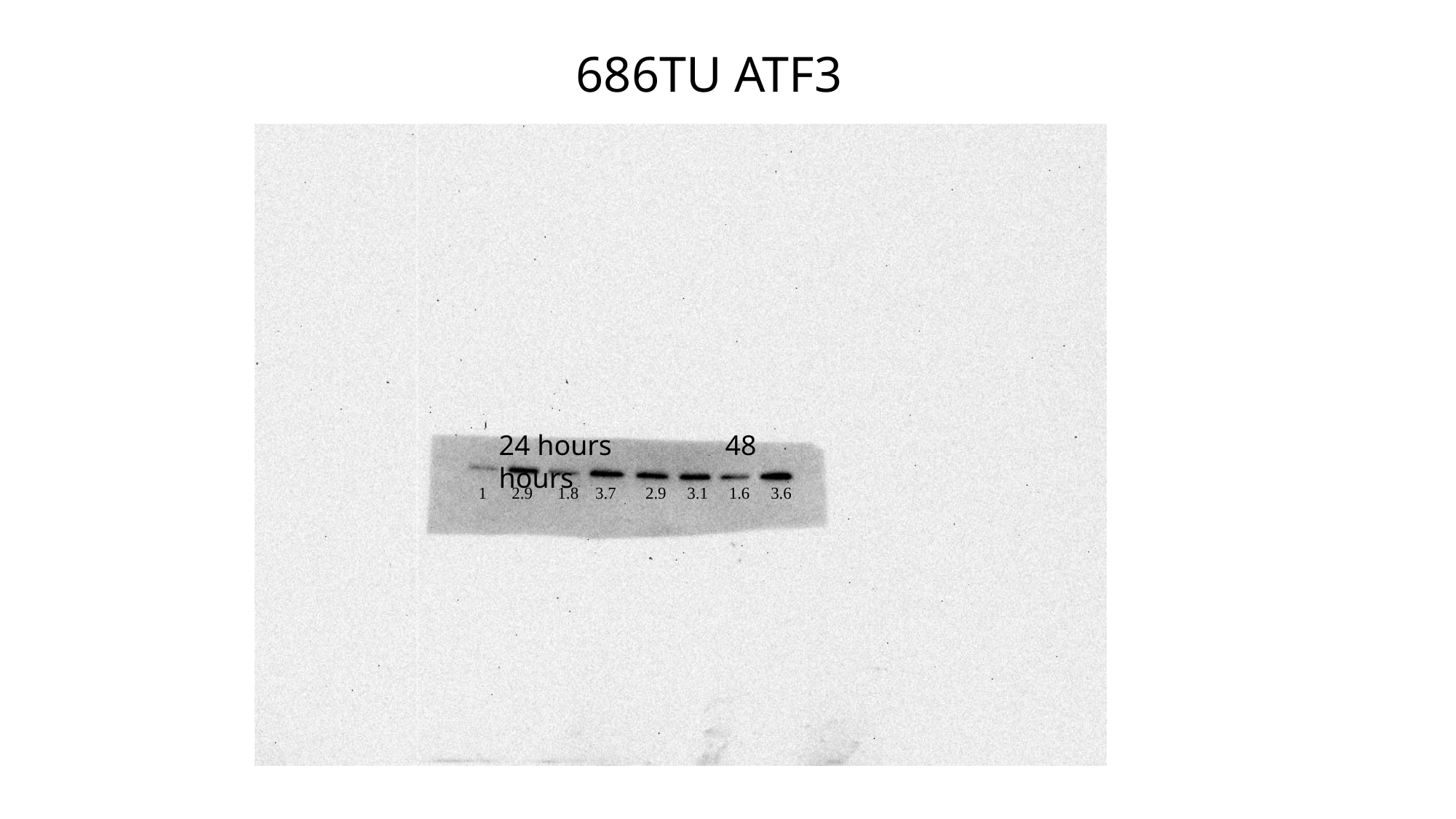

686TU ATF3
24 hours 48 hours
 1 2.9 1.8 3.7 2.9 3.1 1.6 3.6

## Slide 12
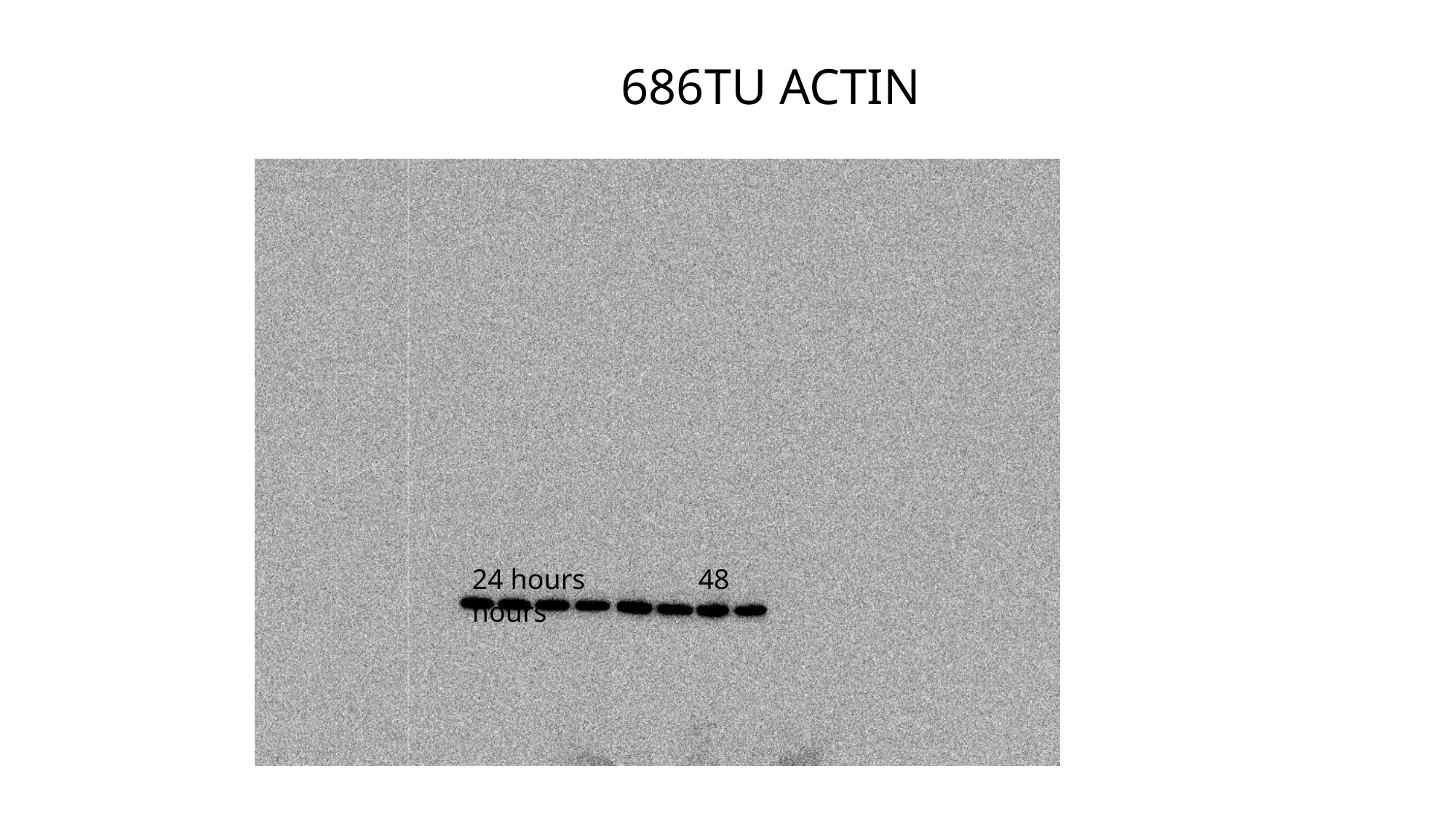

686TU ACTIN
24 hours 48 hours
