# Supplementary material for: Chemoprevention of 4-NQO-Induced Oral Cancer by the Combination of Resveratrol and EGCG: In Vivo, In Silico and In Vitro Studies
Source: Cancers (Basel). 2026 Mar 28;18(7):1098. doi: 10.3390/cancers18071098 (PMC13072290; doi:10.3390/cancers18071098)
Supplement: Supplementary file 1 [file cancers-18-01098-s001.zip › Supplementary Figures.pdf]

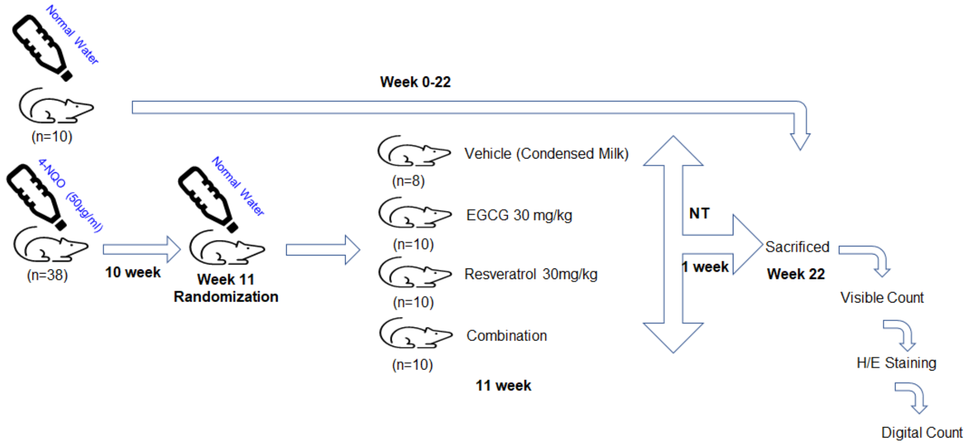

Supplementary Figure S1: Schema for chemoprevention study

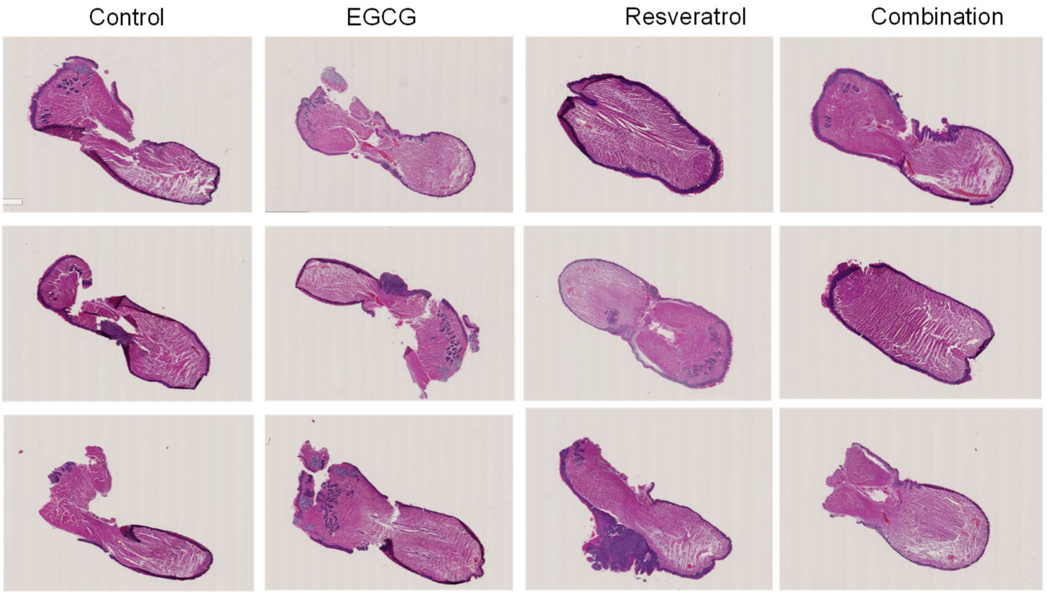

Supplementary Fig. S2: Three H&E stained mice tongues from each group.
